# Sebaceous carcinoma of the breast: a case report

**DOI:** 10.1186/s40792-017-0312-4

**Published:** 2017-02-23

**Authors:** Yuta Yamamoto, Toshitsugu Nakamura, Hiroshi Koyama, Toshiharu Kanai, Suzuko Moritani, Shu Ichihara

**Affiliations:** 1 Department of Surgery, Suwa Red Cross Hospital, Suwa, Japan; 2 Present address: Department of Surgery, Shinshu University School of Medicine, Matsumoto, Japan; 3 Department of Pathology, Suwa Red Cross Hospital, Suwa, Japan; 4 Koyama Clinic, Suwa, Japan; 5 Department of Pathology, National Hospital Organization Nagoya Medical Center, Nagoya, Japan; 6 Present address: Department of Pathology, Shiga University of Medical Science, Otsu, Japan

**Keywords:** Breast carcinoma, Sebaceous carcinoma, Lipid staining, Immunohistochemistry

## Abstract

**Background:**

Sebaceous carcinoma of the breast is a distinct variant of invasive ductal carcinoma. It is rare and only several cases have been reported.

**Case presentation:**

An 80-year-old woman noted bloody discharge from her left nipple and palpated a lump in her left breast. Ultrasonography revealed a 19-mm mass in the left breast. Fine-needle aspiration suggested invasive ductal carcinoma. Partial mastectomy and sentinel lymph node biopsy were performed. On histological examination, the tumor revealed solid growth of small, round uniform cells with clear cytoplasm, partially intermingled with vacuolated cells indicative of sebaceous differentiation. The tumor cells contained abundant Sudan Black B-positive lipid droplets in the cytoplasm, and they were immunohistochemically positive for adipophilin. They were negative for estrogen receptor, progesterone receptor, and androgen receptor; positive for cytokeratin 7 and Ber-EP4; and partially positive for epithelial membrane antigen. Based on these findings, the patient was diagnosed with sebaceous carcinoma of the breast.

**Conclusions:**

We diagnosed a rare case of sebaceous carcinoma of the breast.

## Background

Sebaceous carcinoma is a malignant epithelial tumor derived from or mimicking the sebaceous glands. It is usually found in the eyelid tumor or skin adnexal tumor [1, 2], whereas sebaceous carcinoma of the breast is quite rare and only several cases have been described. We report a rare case of sebaceous carcinoma of the breast and review the immunopathological findings of the cases reported previously.

## Case presentation

**Fig. 1:**
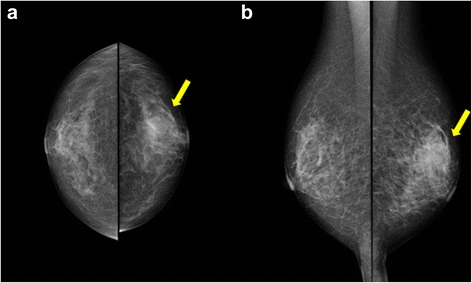
**a, b** Mammogram shows a focal asymmetry in the left CC-O (**a**) and left MLO-M area (**b**) (*arrows*)

**Fig. 2:**
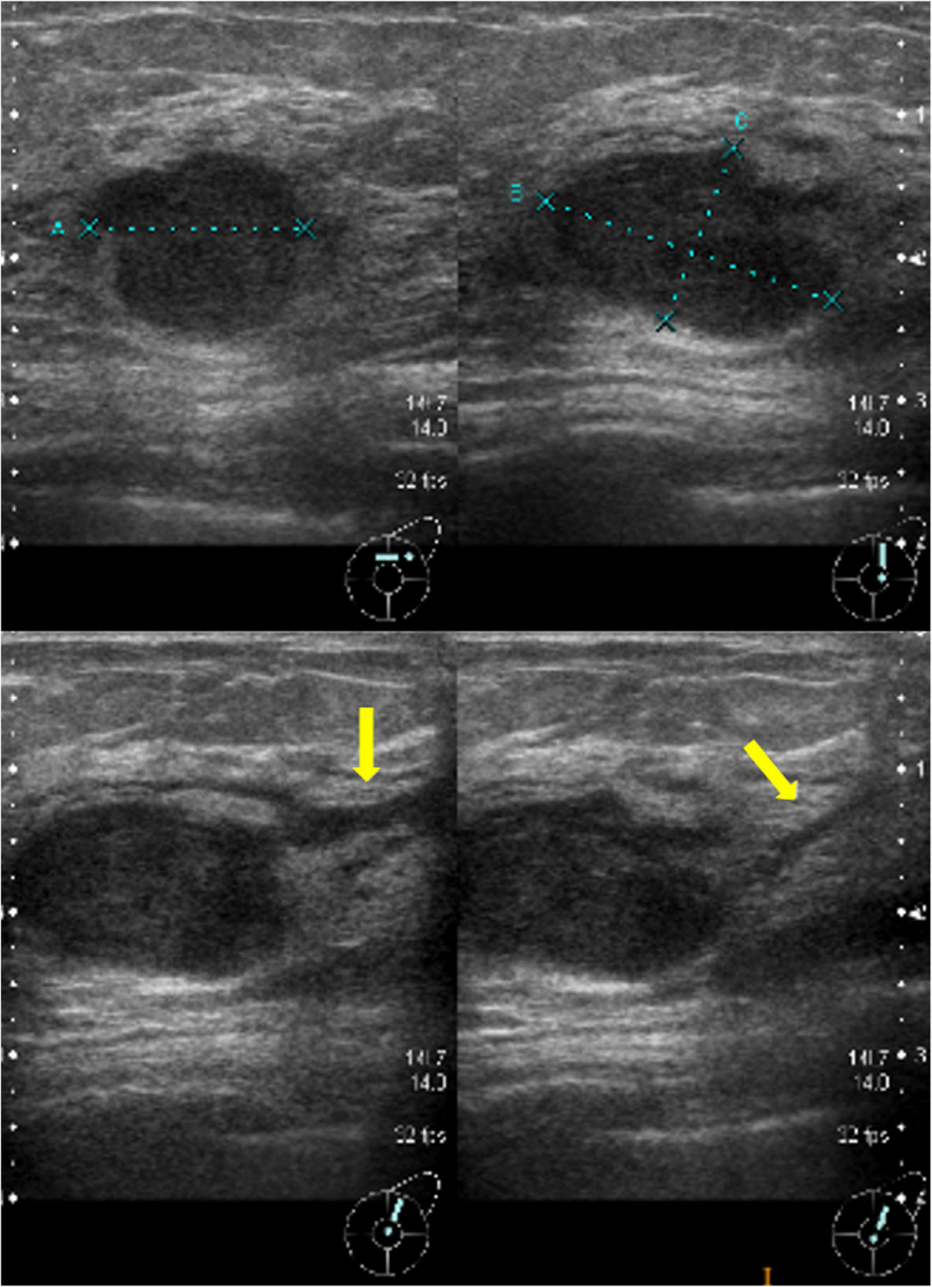
Ultrasonography shows a hypoechoic mass measuring 19 × 17 × 11 mm with an irregular margin, acoustic enhancement, and interruption of the posterior border of the mammary gland in the upper lateral quadrant of the left breast. It has connections with the surrounding mammary ducts (*arrows*)

**Fig. 3:**
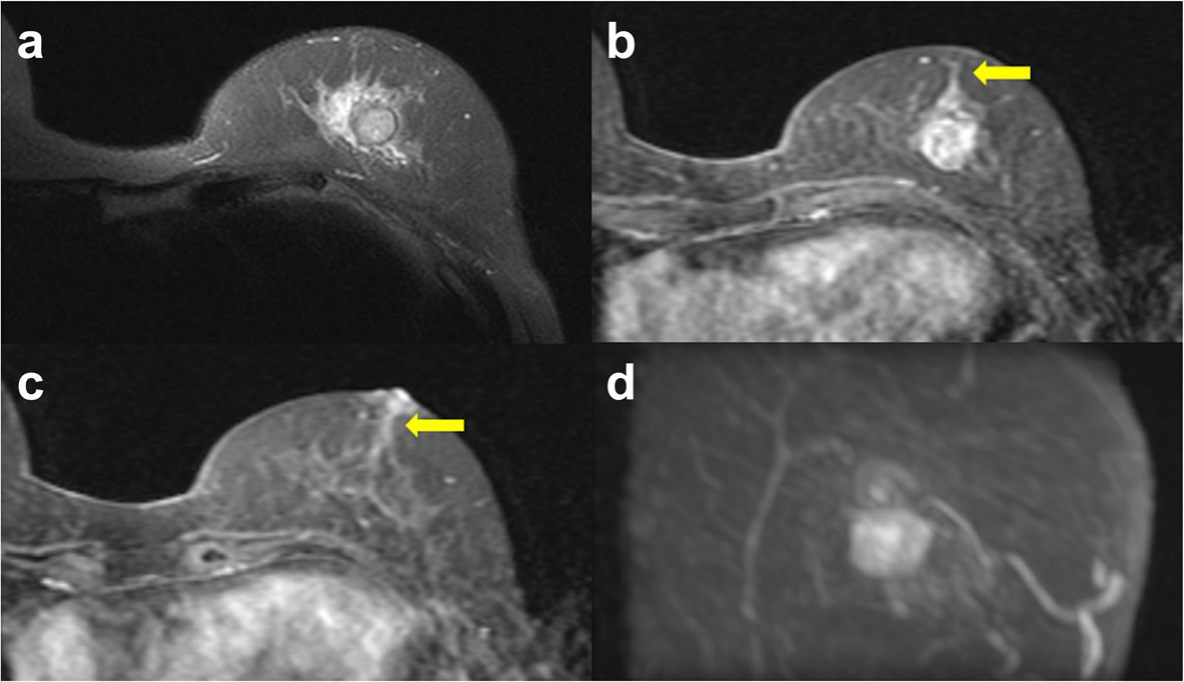
**a** Magnetic resonance imaging (MRI) shows that the mass has a relatively high intensity and has a peritumoral low-intensity capsule-like signal on T2-weighted images. **b**, **c** Contrast-enhanced MRI shows a mass with early arterial enhancement and two areas of linear enhancement between the mass and nipple (*arrows*). **d** Maximum intensity projection (MIP)

**Fig. 4:**
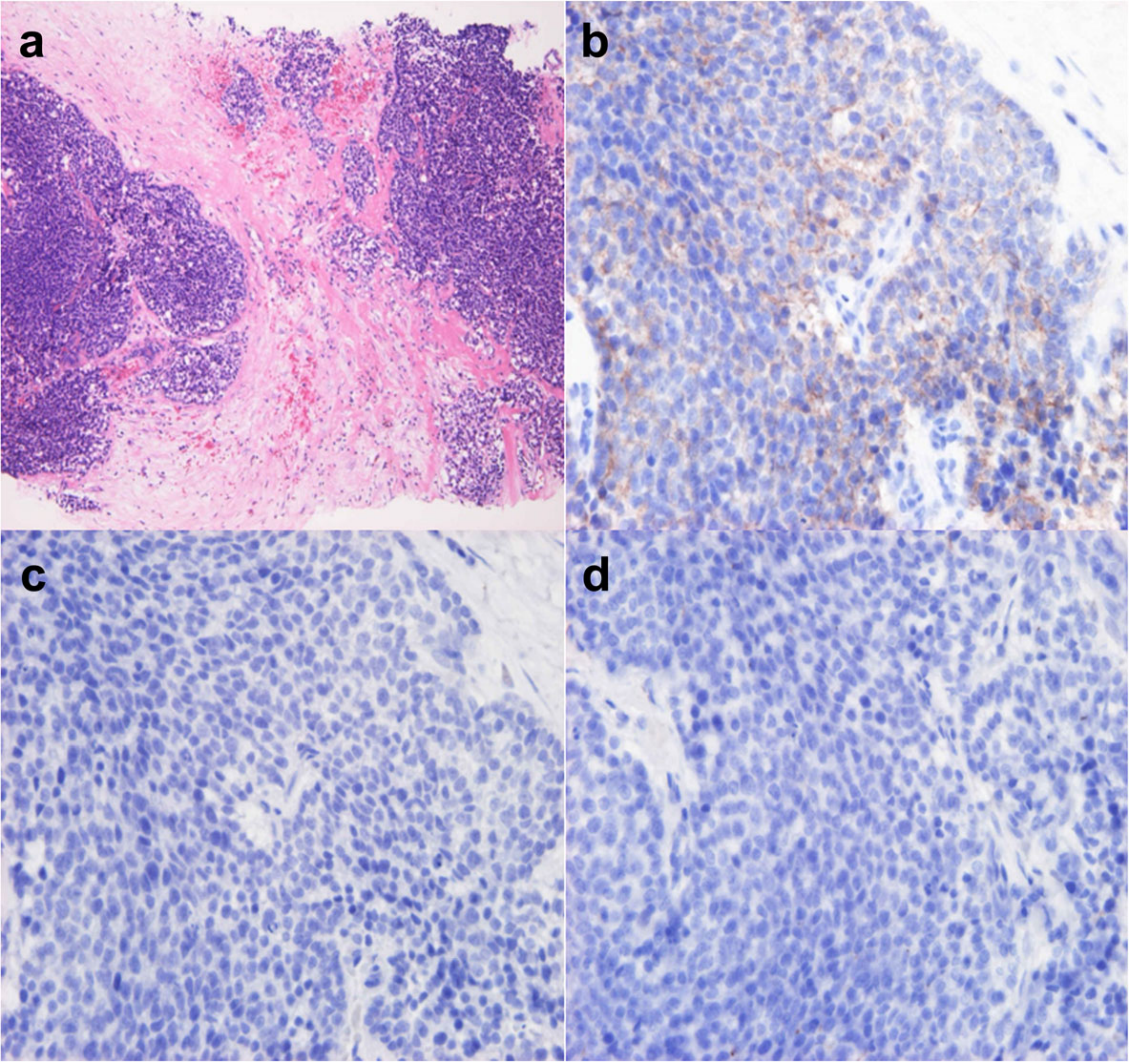
Microscopic findings on core needle biopsy. **a** Core needle biopsy suggests invasive ductal carcinoma (HE stain). **b**–**d** Tumor cells do not express estrogen receptor (ER) (**b**), progesterone receptor (PgR) (**c**), or HER2/neu protein (**d**)

**Fig. 5:**
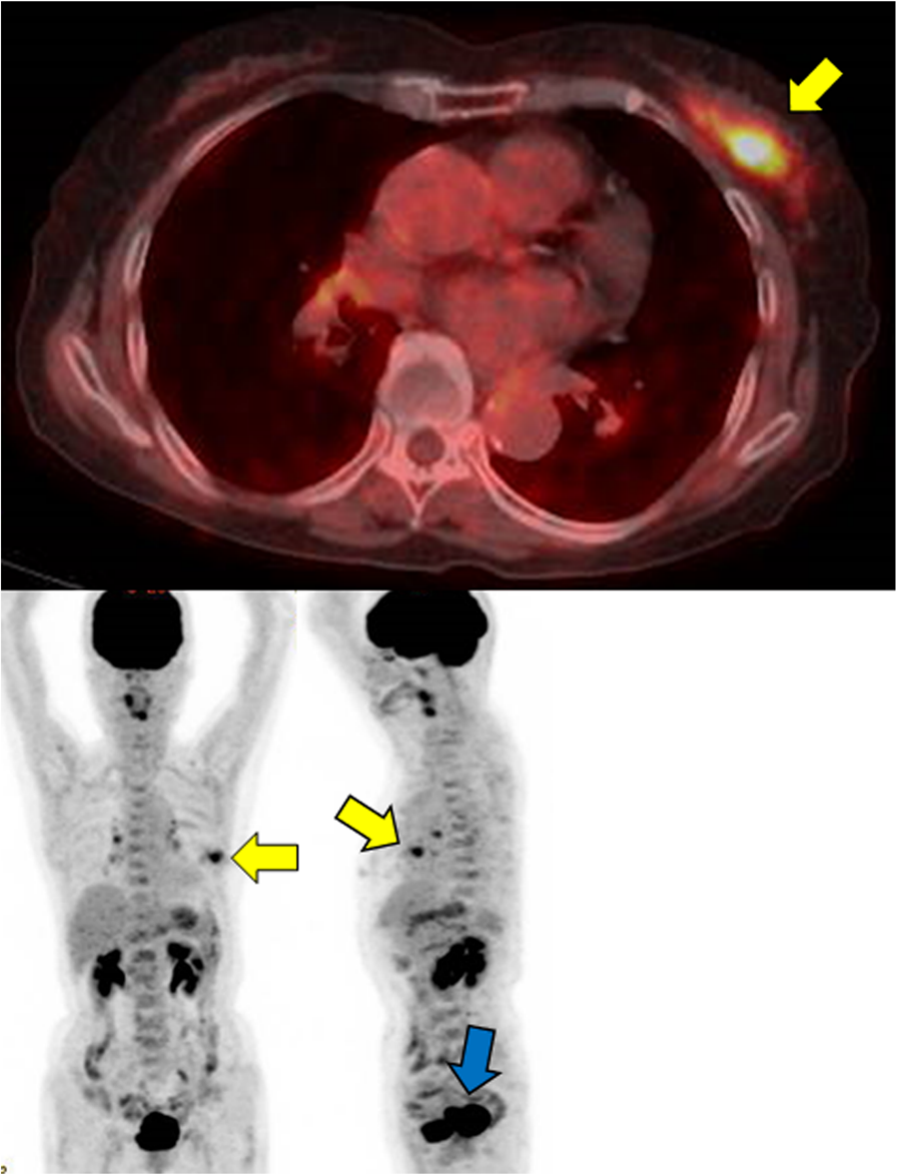
**a**, **b** Whole body fluorodeoxyglucose (FDG) positron emission tomography-computed tomography (PET-CT) images. PET-CT shows a mass in the left breast with a maximum standardized uptake value of 4.4 (*yellow arrows*). **b** It also shows a nodular lesion in the cervix uteri, with high FDG uptake (*blue arrow*)

An 80-year-old woman noticed bloody discharge from her left nipple and palpated a lump in her left breast, and was referred to our hospital. Her mother had a history of breast cancer. Physical examination revealed a hard and immovable mass without skin adhesion in the upper lateral quadrant of the left breast. Cytologically, no malignant cells were found in the bloody discharge from the nipple. Laboratory data on admission were within normal limits. There was no elevation in serum levels of any tumor marker including CEA, CA15-3, NCC-ST-439, ICT, and CA19-9. Mammogram showed a focal asymmetry in the left CC-O (Fig. 1a) and left MLO-M area (Fig. 1b). Ultrasonography revealed a hypoechoic mass measuring 19 × 17 × 11 mm with an irregular margin, acoustic enhancement, and interruption of the posterior border of the mammary gland in the upper lateral quadrant of the left breast. It was connected to the surrounding mammary ducts (Fig. 2). The mammogram and ultrasonography were classified into the Breast Imaging Reporting and Data System (BI-RADS) 5th edition category 3 and 4, respectively. Contrast-enhanced magnetic resonance imaging (MRI) confirmed a mass with early enhancement. The mass had a relatively high intensity, and a peritumoral low-intensity capsule-like signal was noted on T2-weighted images (Fig. 3a). MRI also showed early arterial enhancement, gradual washout, and two lengths of linear enhancement between the mass and nipple (Figs. 3b, c). Core needle biopsy of the lesion demonstrated a solid, proliferating, and invasive tumor with adipose tissue involvement. These findings suggested invasive ductal carcinoma. The tumor was negative for estrogen receptor (ER) (Fig. 4b), progesterone receptor (PgR) (Fig. 4c), and HER2/neu (Fig. 4d). Positron emission tomography-computed tomography (PET-CT) revealed the breast tumor with a maximum standardized uptake value of 4.4 and also showed a 55-mm mass in the cervix uteri (Fig. 5a, b). Biopsy from the latter finally confirmed a diagnosis of cervical carcinoma. After radiotherapy for the cervical carcinoma, the patient underwent partial mastectomy and sentinel lymph node biopsy. She was not treated with neoadjuvant chemotherapy for her triple-negative breast carcinoma due to her great age and advanced cervical carcinoma.

**Fig. 6:**
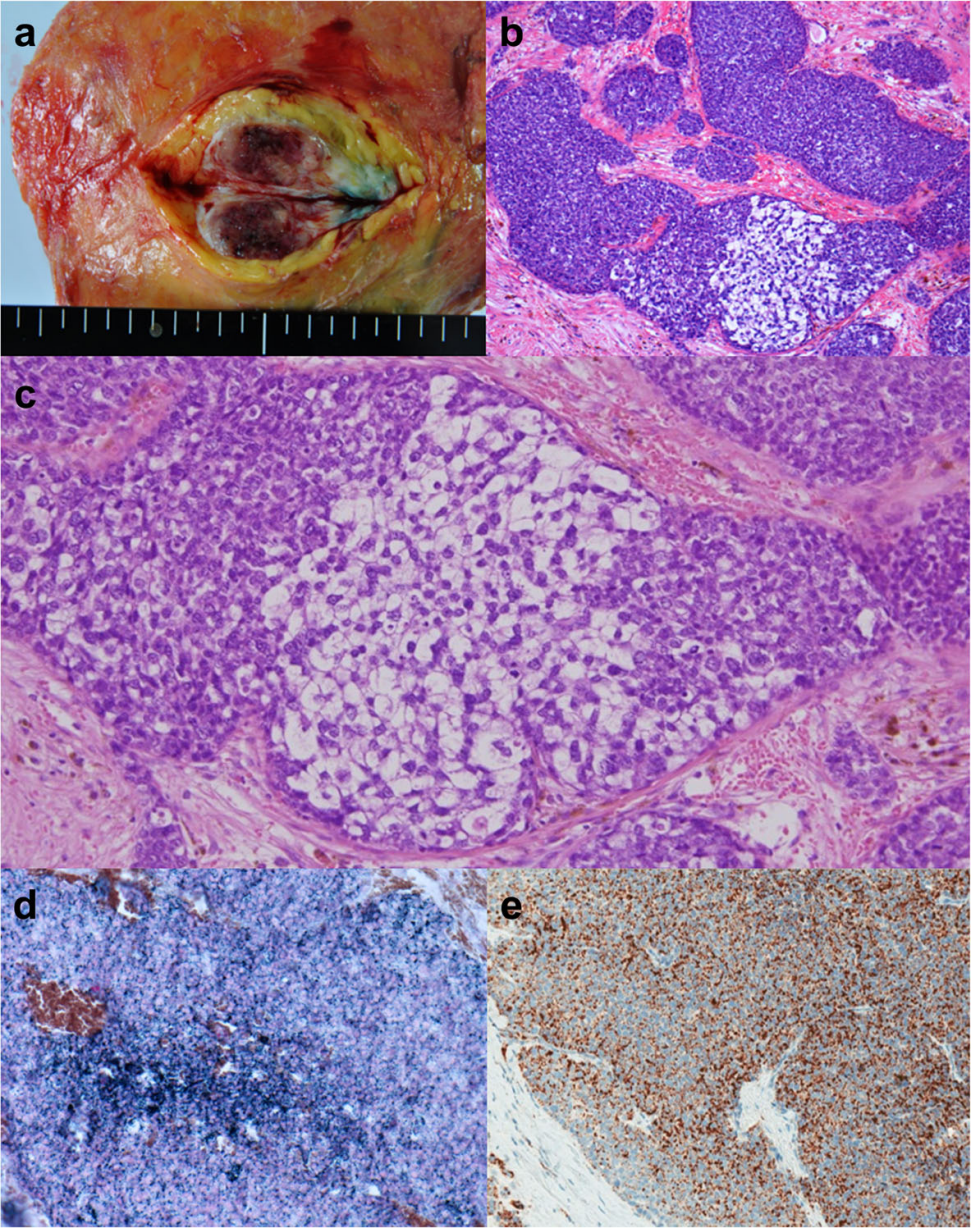
Pathological findings of the resected specimen. **a** Macroscopically, the tumor, measuring 35 mm in its greatest diameter, has no connection to the overlying skin or nipple. **b** The tumor shows solid growth of nests of various sizes in fibrous stroma. c The lesion consists of dense proliferation of relatively small, round cells and partially intermingled with cells with clear cytoplasm, indicating sebaceous differentiation (HE stain). **d** The tumor cells contain abundant Sudan Black B-positive lipid droplets in the cytoplasm. **e** On immunohistochemistry, 90% of the cells are positive for adipophilin

Macroscopically, the breast mass measured 35 mm in its greatest diameter and had no connection to the overlying skin or nipple (Fig. 6a). Histologically, the tumor revealed solid growth of small, round uniform cells with clear cytoplasm. In part, aggregates of large tumor cells with clear and vacuolated cytoplasm, indicative of sebaceous differentiation, were seen (Fig. 6b, c). The intraductal spread of the tumor was observed with bleeding in dilated ducts. A part of tumor nodule was surrounded by the fibrous tissue. No metastatic deposits were identified in the axillary lymph nodes. On frozen sections, most part of the tumor cells contained abundant Sudan Black B-positive lipid droplets in the cytoplasm (Fig. 6d). Immunohistochemically, 90% of the cells were positive for adipophilin (Fig. 6e). The cells were negative for ER, PgR, Her2/neu, and androgen receptor (AR); positive for cytokeratin 7 and Ber-EP4; and partially positive for epithelial membrane antigen (EMA). In addition, the tumor cells were negative for the neuroendocrine markers such as synaptophysin and chromogranin A. Therefore, the present case was diagnosed with sebaceous carcinoma of the breast. She had no complications of surgery and was discharged from the hospital on post-operative day 7.

Sixteen months after her breast surgery, the patient was admitted to the palliative care unit of another hospital due to her metastatic cervical carcinoma. There have been no signs of recurrence of breast malignancy.

### Conclusions

Sebaceous carcinoma of the breast is a distinct variant of invasive ductal carcinoma, characterized by a lobular or nested growth pattern of tumor cells variably admixed with cells displaying sebaceous differentiation [3]. According to the World Health Organization histological classification of tumors of the breast, the definition of the sebaceous carcinoma of the breast is that a primary breast carcinoma of the skin adnexal type with sebaceous differentiation at least 50% of cells and there should be no evidence of derivation from cutaneous adnexal sebaceous glands [4]. The present case fulfilled the necessary condition.

Pathological diagnosis of sebaceous carcinoma of the breast is considered to be difficult when sebaceous differentiation is morphologically obscure. In such cases, it is necessary to show intracytoplasmic lipid by Oil Red O or Sudan Black B stain, or to demonstrate expression of adipophilin by immunohistochemistry. However, lipids are extracted during the organic phase of tissue processing and lipid staining cannot be performed in formalin-fixed paraffin-embedded material. Accordingly, lipid staining can vividly demonstrate intracytoplasmic lipid only when fresh frozen material is available [5]. In the present case, intracytoplasmic lipids were identified through Sudan Black B and the tumor cells were immunohistochemically positive for adipophilin.

Sebaceous carcinoma should be differentiated from other rare types of breast carcinoma composed of vacuolated or clear cells, such as glycogen-rich carcinoma and lipid-rich carcinoma. Glycogen-rich carcinoma can be differentiated easily from sebaceous carcinoma by lack of lipids in the cytoplasm of neoplastic cell. Moreover, neoplastic cells of glycogen-rich carcinoma have a water-clear cytoplasm at light microscopy level, whereas sebaceous carcinoma is composed of vacuolated or foamy cells [6, 7]. Regarding lipid-rich carcinoma, at least 90% of tumor cells have abundant clear or vacuolated lipid-rich cytoplasm [8]. Sebaceous carcinoma shows a compact lobulated solid growth pattern and finely vacuolated cells. In contrast, lipid-rich carcinoma infiltrates like conventional ductal carcinoma and the vacuolization is much less conspicuous.

There is no specific imaging finding that are useful in diagnosis of sebaceous carcinoma of the breast. In MRI of the present case, linear enhancement between mass and nipple and capsule-like signal were observed. The linear enhancement indicates intraductal spread of the tumor. It was identified pathologically with bleeding in dilated ducts that caused bloody discharge from her nipple. The capsule-like signal was pathologically consistent with the fibrous tissue surrounding the tumor nodule. However, they are not specific for sebaceous carcinoma.

The number of previously reported cases of sebaceous carcinoma of the breast is limited, and their clinical and pathological features are available in 12 cases (Table 1) [9–17]. According to the reported cases including ours, 12 patients were women with ages ranging from 25 to 80 years. ER, PgR, and HER2/neu showed positivity in 7 of 12, 8 of 12, and 3 of 9 cases, respectively. Although some hormones or the HER2/neu oncogene may have some role in the development of sebaceous carcinoma, the details are unknown.

**Table 1:** Clinical and pathological features of reported sebaceous carcinoma of the breast

Authors (ref. no.)	Year	Age (years)	Sex	Side	ER	PgR	AR	HER2	Oil red O	Sudan Black B	CAM5.2	CK7	Ber-EP4	EMA	MIB-1	Adipophilin	pTNM^a^	Prognosis
Prescott et al [9]	1992	74	F	R					(+)		(+)			(+)			T2NxMx	NA
Mazzella et al. [10]	1995	55	M	L	(+)	(+)			(+)								T2N0Mx	AWNED, 10 months
Tavassoli [11]	1999	46	F	R	(−)	(+)									20%		T3N0Mx	NA
Varga et al. [12]	2000	45	F	R	(+)	(+)		(−)							16%		T2NxMx	AWD, 132 months
Hisaoka et al. [13]	2006	71	F	R	(+)	(+)			(+)					(+)	38%		T1cN1-2 Mx	NA
Numoto et al. [14]	2007	49	F	L	(+)	(+)	(+)	(−)			(+)			(+)	15%		T1cNxMx	NA
Murakami et al. [15]	2009	50	F	L	(−)	(−)	(+)	(+)			(+)			(+)	30%	(+)	T1cN1-2 Mx	AWNED, 24 months
Ramljak et al. [16]	2010	85	F	L	(−)	(−)		(−)							25%		T2NxMx	NA
Švajdler et al. [17]	2015	65	F	R	(+)	(+)		(−)						(+)	30%		T1cN1aM0	AWNED, 27 months
Švajdler et al. [17]	2015	61	F	R	(−)	(−)		(+)						(+)	80%		T2N1aM1	DOD, 28 months
Švajdler et al. [17]	2015	66	F	R	(+)	(+)		(+)							5%		T2N1aM1	AWD, 70 months
Švajdler et al. [17]	2015	25	F	R	(+)	(+)		(−)						(+)			TxN0Mx	AWNED, 75 months
Yamamoto et al. (current case)		80	F	L	(−)	(−)	(−)	(−)		(+)		(+)	(+)	(+)		(+)	T2N0M0	AWNED, 16 months

*Abbreviations*: *AR* androgen receptor, *AWD* alive with disease, *AWNED* alive with no evidence of disease, *Ber-EP4* EpCAM antibody, *CAM5.2* anti-low molecular weight keratin, *CK7* cytokeratin 7, *DOD* died of disease, *EMA* epithelial membrane antibody, *ER* estrogen receptor, *F* female, *HER2* human epidermal growth factor receptor type 2, *L* left, *M* male, *MIB-1* anti-human Ki-67 antigen, *NA* not available, *PgR* progesterone receptor, *R* right

^a^According to International Union Against Cancer TNM Classification of Malignant Tumors (7th Edition)

It has been reported that sebaceous carcinoma of the breast might be high-grade malignant neoplasm because three of four patients had axillary lymph node metastases and two patients experienced an aggressive clinical course with distant metastases [17]. On the other hand, seven eighths of the cases whose prognoses were available was alive with or without evidence of breast malignancy after operation. The clinical course is not generally known due to its rarity. Further study is warranted to elucidate the pathology and prognosis of the patients with sebaceous carcinoma of the breast.

## Abbreviations

AR: Androgen receptor
Ber-EP4: EpCAM antibody
BI-RADS: Breast Imaging Reporting and Data System
CAM5.2: Anti-low molecular weight keratin
CK7: Cytokeratin 7
EMA: Epithelial membrane antigen
ER: Estrogen receptor
HER2/neu: Human epidermal growth factor receptor type 2
MIB-1: Anti-human Ki-67 antigen
MRI: Magnetic resonance imaging
PET-CT: Positron emission tomography-computed tomography
PgR: Progesterone receptor
